# A risk prediction model for acute kidney injury in patients with pulmonary tuberculosis during anti-tuberculosis treatment

**DOI:** 10.1080/0886022X.2022.2058405

**Published:** 2022-04-04

**Authors:** Zhi Xiang Du, Fang Qun Chang, Zi Jian Wang, Da Ming Zhou, Yang Li, Jiang Hua Yang

**Affiliations:** a Department of Infectious Diseases, Taizhou People's Hospital, Taizhou, China; b Department of Geriatric respiratory and critical illness, The First Affiliated Hospital of Anhui Medical University, Hefei, China; c Department of Infectious Diseases, Yijishan Hospital, Wannan Medical College, Wuhu, China

**Keywords:** Risk prediction model, pulmonary tuberculosis, acute kidney injury, nomogram

## Abstract

**Background:**

Acute kidney injury (AKI) is not a rare complication during anti-tuberculosis treatment in some patients with pulmonary tuberculosis (PTB). We aimed to develop a risk prediction model for early recognition of patients with PTB at high risk for AKI during anti-TB treatment.

**Methods:**

This retrospective cohort study assessed the clinical baseline, and laboratory test data of 315 inpatients with active PTB who were screened for predictive factors from January 2019 to June 2020. The elements were analyzed by logistic regression analysis. A nomogram was established by the results of the logistic regression analysis. The prediction model discrimination and calibration were evaluated by the concordance index (C-index), ROC curve, and Hosmer-Lemeshow analysis.

**Results:**

A total of 315 patients with PTB were enrolled (67 patients with AKI and 248 patients without AKI). Seven factors, including microalbuminuria, hematuria, cystatin-C (CYS-C), albumin (ALB), creatinine-based estimated glomerular filtration rates (eGFRs), body mass index (BMI), and CA-125 were acquired to develop the predictive model. According to the logistic regression, microalbuminuria (OR = 3.038, 95%CI 1.168–7.904), hematuria (OR = 3.656, 95%CI 1.325–10.083), CYS-C (OR = 4.416, 95%CI 2.296–8.491), and CA-125 (OR = 3.93, 95%CI 1.436–10.756) were risk parameter, while ALB (OR = 0.741, 95%CI 0.650–0.844) was protective parameter. The nomogram demonstrated good prediction in estimating AKI (C-index= 0.967, AUC = 0.967, 95%CI (0.941–0.984), sensitivity = 91.04%, specificity = 93.95%, Hosmer-Lemeshow analysis SD = 0.00054, and quantile of absolute error = 0.049).

**Conclusions:**

Microalbuminuria, hematuria, ALB reduction, elevated CYS-C, and CA-125 are predictive factors for the development of AKI in patients with PTB during anti-TB treatments. The predictive nomogram based on five predictive factors is achieved good risk prediction for AKI during anti-TB treatments.

## 1. Introduction

Tuberculosis (TB) is a common infectious disease with a long history in China and is still one of the top 10 causes of death worldwide. The death rate of TB is still higher than that of HIV. According to the WHO Global Tuberculosis Report, approximately 1.7 billion people are infected with *Mycobacterium tuberculosis* (*M. tuberculosis*), and 10 million people are diagnosed with TB each year worldwide [1]. Although modern chemotherapy has played a pivotal role in combating TB, adverse drug events limit the completion rate of treatment [2].

Acute kidney injury (AKI) is not a single disease entity, and it is defined as an abrupt decline in kidney function that occurs within 7 days or less. An epidemiological survey estimated that 2 million people worldwide die of AKI every year, whereas some patients with AKI are at increased risk of developing chronic kidney disease (CKD) [3]. The retrospective study indicated that AKI is usually observed in patients admitted to the intensive care unit (ICU), and half of all such AKI was septic AKI [4]. In other studies, only one-third of cases of AKI were hospital-acquired, and the rest were community-acquired. Through statistical verification, increased GFR and proteinuria are independent risk factors for AKI [5].

AKI generally occurs in patients with acute or chronic diseases. In ICU patients, the predominant etiological category of AKI is sepsis, and the most common causes of sepsis are gram-negative bacteria, and fungal, etc [6]. A 5-year retrospective study indicated that AKI is not a rare complication during anti-TB treatment in the elderly population [7]. Acute kidney injury (AKI), acute kidney disease (AKD), and chronic kidney disease (CKD) can form a process of continuous kidney injury. A diagnosis of AKI should evaluate the possible cause of persistent kidney injury. Several studies have confirmed that a hypersensitivity reaction induced by rifampin is the leading cause of AKI during anti-TB treatment. Serum creatinine usually increases within 2 months of anti-TB-treatment and returns to baseline within 3 months. At the same time, approximately 27% of patients with AKI will develop permanent kidney damage [8]. More importantly, AKI is asymptomatic, so screening and prediction of kidney injury during anti-TB treatment are essential.

Kidney Disease: Improving Global Outcomes (KDIGO) recommends stratified screening of AKI occurrence based on the exposed and susceptive factors. However, there are few instruments to predict the occurrence of AKI [3].

In recent years, risk prediction models have been increasingly used to estimate the probability of occurrence (diagnostic models) or outcomes (predictive models) of a particular disease [9]. In Guan Chen’s study, predictive factors such as age, previous surgical history, and cardiac arrhythmia were used to establish a prediction model to predict cardiac surgery-associated acute kidney injury (CSA-AKI). This model can improve the identification of patients at high risk for AKI before cardiac surgery [10]. Machine learning was used to develop risk prediction models in recent study [11]. Unfortunately, no prediction models have been developed to predict the risk of AKI during anti-TB treatment.

This study divided patients with pulmonary tuberculosis (PTB) into two groups according to AKI occurrence during anti-TB treatment. Clinical baseline and laboratory data were compared to obtain the different factors and establish a prediction model. The aim of this study was to develop a risk prediction model to effectively assess the risk of AKI before anti-TB treatment to prevent renal injury.

## 2. Materials and methods

### 2.1. Study population

This article was a retrospective observational study. This study obtained approval from the Clinical Research Ethics Committee of Taizhou People's Hospital TZRY-LL-AF/SQ-014-2.0 (protocol number KY201803901). All tuberculosis diagnoses and treatments for TB were conformed to the ‘Guidelines for Diagnosing and Treating Tuberculosis (2001)’ [12].

**Figure 1.**
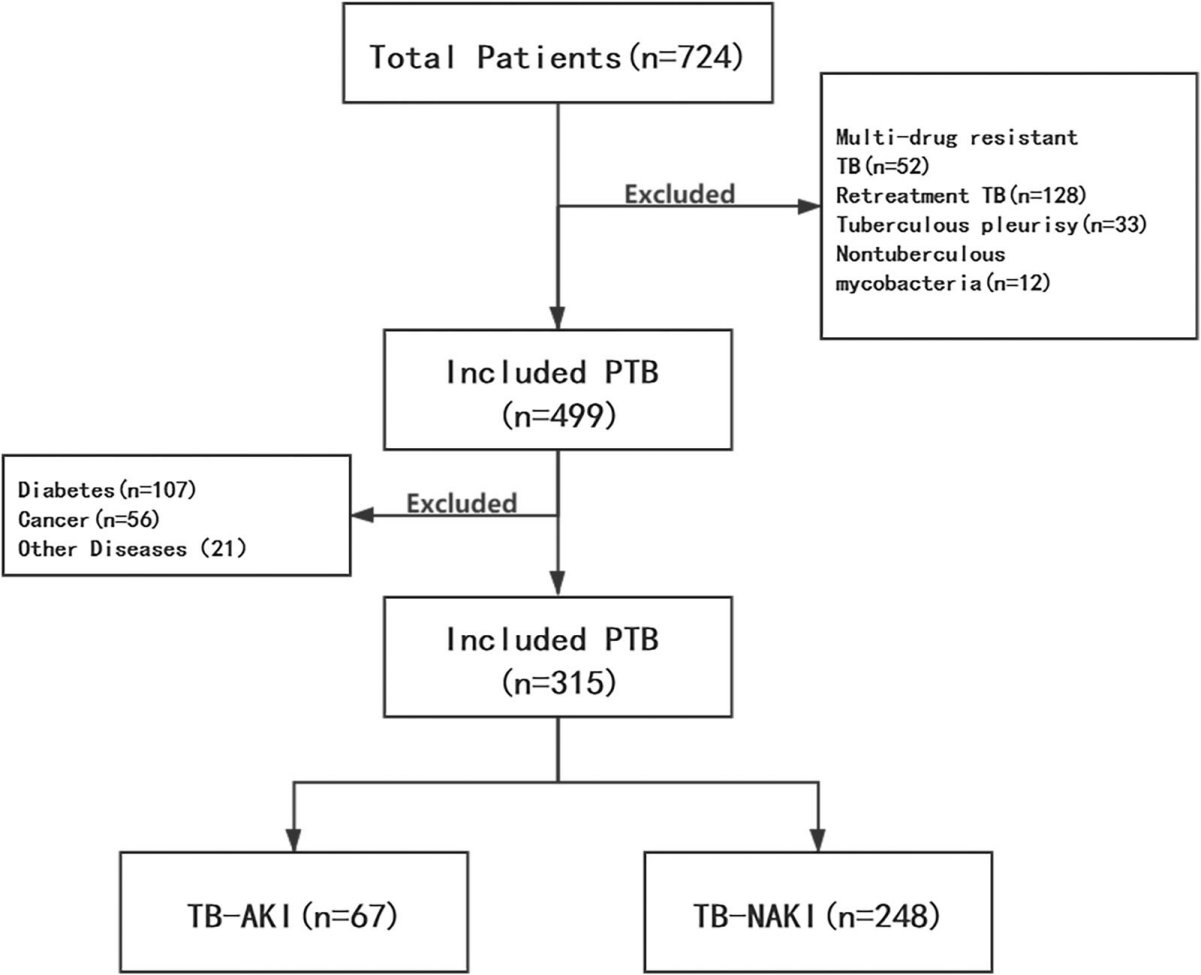
Flow chart of patient inclusion and exclusion (PTB: pulmonary tuberculosis; TB-AKI: pulmonary tuberculosis with acute kidney injury; TB-NAKI: pulmonary tuberculosis without acute kidney injury).

A total of 724 individuals were enrolled at the Department of Infectious Diseases, Taizhou People’s Hospital, Jiangsu Province between January 2019 and June 2020. According to the inclusion and exclusion criteria, 52 patients with Multidrug-resistant TB, 128 patients with retreatments of PTB, 33 patients with tuberculous pleurisy, 12 patients with nontuberculosis mycobacteria patients and 184 patients with the underlying disease were excluded. Finally, 315 patients with primary PTB were eventually enrolled in the statistical comparison (Figure 1).

Inclusion criteria: 1. According to the WHO guidelines, all patients with PTB had typical imaging lesions, positive sputum smears, or positive sputum cultures of Mycobacterium tuberculosis; 2. Acute kidney injury (AKI) was diagnosed by the KDIGO criteria established in 2012, serum creatinine increase ≥0.3 mg/dl (≥26.5 μmol/L) within 48 h, or serum creatinine increase ≥50% from the baseline value within 7 days or duration of decrease in urine output (urine volume <0.5) ≥ 6 h [13].

Exclusion criteria: 1. Patients who had received anti-TB treatment before admission; 2. Patients diagnosed with multidrug-resistant TB, tuberculous pleurisy, retreatment of PTB, and nontuberculous mycobacterial infection; 3. PTB Patients who had underlying diseases such as hypertension, diabetes, immune diseases, kidney diseases, malignant tumors, viral hepatitis etc were excluded from enrollment.

All patients with active PTB were given standardized anti-TB treatment consisting of oral rifampicin (R) 10 mg/kg/d, isoniazid (H) 5 mg/kg/d, pyrazinamide (Z) 25 mg/kg/d, and ethambutol (E) 20 mg/kg/d for 2 months, followed by daily isoniazid and rifampicin for 4 months (2HRZE/4HR). The regimen was modified by the infectious disease physician, if necessary, when there were adverse drug effects.

### 2.2. Data Collection and study design

The enrolled patients were divided into two groups, the AKI-group: PTB with AKI and the control group, PTB without AKI.

The different factors were obtained from the clinical baseline and laboratory test data of the two groups. The clinical baseline included age, sex, occupation, residence, and lung imaging characteristics, etc. Laboratory test data included routine urine (PH value, UM/urine microalbumin, URBC/hematuria, etc.); Blood routine (RBC, NEU, etc.); liver function (TBIL, ALB, ALT, AST, etc.) and kidney function (BUN, CREA, UA, B2MG, CYS-C, etc.). Tumor and inflammatory indicators were also included in the laboratory test data. The CA-125, CEA, and CA19-9 cutoff points were selected to be 22 U/ml, 5.0 ng/ml, and 37 U/L, respectively. The liver and kidney function tests were reviewed within 7 days after anti-TB treatment.

The predictive factors for AKI in patients with PTB during anti-TB treatment were analyzed by logistic regression analysis. A prognostic nomogram was established with the results of the logistic regression analysis.

The immunological indices of the two groups were compared to explore the possible immunological mechanisms of AKI. Abbreviations in the text are explained in the Abbreviations section.

### 2.3. Statistical analysis

The data were expressed as the frequencies (n), percentages (%), means ± standard deviations (SDs), and median (interquartile ranges) and were analyzed with R version 4.0.2. Count data were analyzed with the chi-square test. If measurement data conformed to a normal distribution, the *t*-test was used to analysis. The Mann–Whitney U-test was used to compare nonnormally distributed data. Before regression analysis, the factors were verified by multiple linear regression to verify independence. Continuous variables (CYS-C ALB, eGFR, and BMI) were substituted into the model according to covariates; categorical variables (UM, URBC, and CA-125) were substituted into the model according to categorical covariates. The nomogram was constructed based on the results of the logistical regression results using the rms package in R software. The discrimination of the prediction model was verified by the C-index and the receiver-operating characteristic curve (ROC curve). The calibration curve verified the correctness of the prediction model. *p* < .05 was considered statistically significant.

## 3. Results

### 3.1. Characteristics of clinical baseline data

A total of 315 patients who received initial treatment for pulmonary tuberculosis patients (PTB) were enrolled in this study. 67 (21.27%) patients with PTB who had acute kidney injury were included in the AKI group, and 248 (78.73%) patients with PTB who did not have AKI were included in the control group; There was no significant difference between the two groups in age, sex, occupation, or the extent of lesion involvement (*p* > .05);

The average BMI in the control group was 19.99 ± 2.82 kg/m2, which was significantly higher than that in the AKI-group 17.90 ± 2.39 kg/m2(*p* < .05); The positive proportion accounted for CA-125 was 35.89% in the control group, which was significantly lower than that in the AKI group 83.87% (*p* < .05); There was no significant difference in T-SPOT, CEA, CA199, CRP, or ESR between the two groups (Table 1).

**Table 1.** The clinical baseline data of PTB patients.

Variable	Control Group	AKI Group	Total	Statistics(χ^2^/t/z)	*p-value*
**n**	248(78.73%)	67(21.27%)	315		
**Age**(year)	56.68 ± 18.77	58.36 ± 19.22	57.03 ± 18.85	0.647	.518
**Sex**					
Male	182(73.39%)	51(76.12%)	233(73.97%)	0.205	.754
Female	66(26.61%)	16(23.88%)	82(26.03%)		
**BMI**(kg/m^2^)	19.99 ± 2.82	17.90 ± 2.39	18.95 ± 2.61	5.544	<.001
**Occupation**					
Individual	5(2.02%)	1(1.49%)	6(1.90%)	11.659	.234
Worker	17(6.85%)	3(4.48%)	20(6.35%)		
Medical staff	2(0.81%)	1(1.49%)	3(0.95%)		
Farmer	56(22.58%)	15(22.39%)	71(22.54%)		
Retirement	61(24.60%)	20(29.85%)	81(25.71%)		
Student	13(5.24%)	1(1.49%)	14(4.44%)		
Staff	40(16.13%)	6(8.96%)	46(14.60%)		
Free	11(4.44%)	1(1.49%)	12(3.81%)		
Other	4(1.61%)	2(2.99%)	6(1.90%)		
Unemployed	37(14.92%)	19(28.36%)	56(17.78%)		
**Lesion site**				1.741	.419
Two lungs	182(80.60%)	54(80.60%)	236(74.92%)		
Left lung	26(5.97%)	4(5.97%)	30(9.52%)		
Right lung	40(13.43%)	9(13.43%)	49(15.56%)		
**T-SPOT**				0.645	.422
(+)	142(57.26%)	42(62.69%)	184(58.41%)		
(−)	106(42.74%)	25(37.31%)	131(41.59%)		
**CEA**				2.928	.087
(+)	12(10.45%)	7(10.45%)	19(6%)		
(−)	236(89.55%)	60(89.55%)	296(96.97%)		
**CA-125**				37.141	<.001
(+)	89(35.89%)	52(83.87%)	141(44.76%)		
(−)	159(64.11%)	15(16.13%)	174(55.24%)		
**CA19-9**				0.463	.291
(+)	39(19.40%)	13(19.40%)	52(16.51%)		
(−)	209(80.60%)	54(80.60%)	263(83.49%)		
**CRP**(mg/l)	19.23 (3.18–46.94)	47.23 (11.73–86.54)	23.86 (5.11–54.04)	1.816	.178
**ESR**(mm/h)	32.68 ± 30.38	48.92 ± 34.70	40.08 ± 32.54	1.103	.294

CEA: carcinoembryonic antigen; CA-125: cancer antigen 125; CA19-9: cancer antigen 19-9; T-SPOT: T-SPOT.TB; CRP: C-reactive protein; ESR: erythrocyte sedimentation rate; BMI: body mass index. Statistical method: chi-square test, *t*-test, Mann-Whitney U-test.

### 3.2. Characteristics of routine blood and urine tests

The positive proportion of microalbuminuria (UM) was 17.74% in the control group, which was significantly lower than that in the AKI group 61.19% (*p* < .05). The number of patients with hematuria in the control group was 26 (10.48%), which was significantly lower than 33(49.25%) in the AKI group (*p* < .05) (Table 2).

**Table 2.** The characteristics of blood routine and urine routine in PTB patients.

Variable	Control Group	AKI Group	Total	Statistics (χ^2^/t/z)	*p-value*
**n**	248 (78.73%)	67 (21.27%)	315		
**RBC** (10^9^/L)	4.22 ± 2.43	3.89 ± 0.71	4.148 ± 2.186	1.080	.281
**HB** (g/L)	118.54 ± 21.23	122.58 ± 19.45	120.56 ± 20.34	1.406	.161
**NEU** (10^9^/L)	5.05 ± 2.59	5.55 ± 2.18	5.30 ± 2.39	1.435	.152
**LYM** (10^9^/L)	1.025 (0.74–1.5)	0.95 (0.62–1.25)	0.99 (0.73–1.44)	1.814	.700
**MON** (10^9^/L)	0.52 (0.37–1.5)	0.55 (0.39–0.83)	0.52 (0.37–0.72)	0.829	.407
**PLT** (10^9^/L)	232.5 (182.00–303.00)	219.00 (171.00–327.00)	232.00 (178.00–307.00)	0.100	.921
**USG**	1.02 (1.013–1.025)	1.02 (1.015–1.025)	1.02 (1.014–1.025)	0.680	.497
**Urine PH**	6.24 ± 0.7	6.16 ± 0.72	6.22 ± 0.706	0.856	.393
**UM**				50.549	<.001
** (+)**	44 (17.74%)	41 (61.19%)	85 (26.98%)		
** (−)**	204 (82.26%)	26 (38.81%)	230 (73.02%)		
** UCR** (g/L)	1.00 (0.5–4.4)	1.00 (1.00–5.00)	1.00 (0.5–4.4)	1.146	.252
**Hematuria**				52.088	<.001
** (+)**	26 (10.48%)	33 (49.25%)	59 (18.73%)		
** (−)**	222 (89.52%)	34 (50.75%)	256 (81.25%)		

RBC: red blood cell; HB: hemoglobin; NEU: neutrophile granulocyte; LYM: lymphocyte; MON: monocyte; PLT: blood platelet; USG: Urine specific gravity; UM: microalbuminuria; UCR: Urine creatinine. Statistical methods: independent sample *t*-test, nonparametric test, chi-square test.

### 3.3. Characteristics of liver and kidney function tests

With regard to liver and kidney function tests, the ALB level in the AKI group was 28.94 ± 3.46 g/l, which was significantly lower than 35.17 ± 4.8 g/l in the control group (*p* < .05); The level of CYS-C in the AKI group was 1.66 ± 0.51 mg/l, which was significantly higher than 0.8 ± 0.43 mg/l in the control group (*p* < .05); The eGFR level in the AKI group was 83.50 ± 36.98 mL/min per1.73m2, which was lower than 94.61 ± 35.34 mL/min per1.73m2 in the control group; There was no significant difference in other indices between the two groups (Table 3).

**Table 3.** The characteristics of liver and kidney function in PTB patients.

Variable	Control Group	AKI Group	Total	Statistics (t/z)	*p-value*
**N**	248 (78.73%)	67 (21.27%)	315		
**TBIL** (umol/L)	10.15 (7.60–13.25)	10.30 (8.10–13.10)	10.30 (7.70–13.10)	1.001	.317
**DBIL** (umol/L)	2.62 ± 1.11	2.89 ± 0.94	2.76 ± 1.03	1.829	.069
**TP** (g/L)	66.34 ± 9.31	65.63 ± 12.33	66.19 ± 10.01	0.511	.609
**ALB** (g/L)	35.17 ± 4.8	28.94 ± 3.46	33.85 ± 5.21	4.959	<.001
**ALT** (U/L)	12.00 (8.00–20.00)	12.00 (8.00–20.00)	12.00 (8.00–20.00)	0.467	.641
**AST** (U/L)	22.04 ± 10.62	22.31 ± 8.09	22.18 ± 9.36	0.193	.847
**ALP** (U/L)	95.27 ± 37.12	104.61 ± 56.09	97.62 ± 41.96	1.621	.106
**GGT** (U/L)	24.00 (16.00–24.00)	25.00 (18.00–46.00)	24.00 (17.00–42.00)	1.612	.107
**LDH** (U/L)	183.03 ± 48.28	193.31 ± 71.56	185.22 ± 54.35	1.376	.170
**CK** (U/L)	42.5 (30.00–65.75)	41.00 (28.00–83.00)	42.00 (30.00–66.00)	0.499	.618
**ACE** (U/L)	31.89 ± 12.62	33.4 ± 17.25	32.21 ± 13.72	0.800	.424
**HCY** (umol/L)	12.21 ± 5.59	14.2 ± 11.13	12.63 ± 7.16	2.030	.161
**BUN** (mmol/L)	4.81 ± 2.00	5.26 ± 2.58	4.91 ± 2.14	1.544	.124
**CREA** (umol/L)	63.81 ± 29.87	69.73 ± 54.60	65.07 ± 36.52	1.179	.239
**UA** (umol/L)	287.87 ± 108.07	269.62 ± 96.54	283.99 ± 105.84	1.253	.184
**β_2_MG** (mg/L)	2.38 ± 0.83	2.48 ± 0.82	2.43 ± 0.83	0.894	.372
**CYS-C** (mg/L)	0.8 ± 0.43	1.66 ± 0.51	0.98 ± 0.57	14.110	<.001
**eGFR** (ml/min per 1.73m^2^)	94.61 ± 35.34	83.50 ± 36.98	89.06 ± 36.16	2.259	.025

TBIL: total bilirubin; DBIL: direct bilirubin; TP: total protein; ALB: albumin; ALT: alanine aminotransferase; AST: aspartate aminotransferase; ALP: serum alkaline phosphatase; GGT: gamma-glutamyl transpeptidase; LDH: lactate dehydrogenase; CK: creatine kinase; ACE: angiotensin converting enzyme; HCY: homocysteine; BUN: urea nitrogen; CREA: creatinine; UA: uric acid; β2MG: β2-microglobulin; CYS-C: cystatin-C; eGFR: estimated glomerular filtration rate. Statistical methods: independent sample *t*-test, nonparametric test.

### 3.4. The renal function characteristics of PTB-AKI patients before and after treatment

In 67 patients with AKI-TB, the five renal functions BUN, CREA, UA, B2MG, and CYS-C, were significantly increased after anti-TB treatment compared with before treatment (Table 4).

**Table 4.** The characteristics of the renal function of PTB-AKI patients before and after treatment.

Values	Before-T	After-T	Statistics(t)	*p-value*
**N**	67	67		
**BUN** (mmol/L)	5.26 ± 2.58	10.61 ± 5.00	9.107	<.001
**CREA** (umol/L)	69.74 ± 54.60	165.88 ± 104.83	11.698	<.001
**UA** (umol/L)	269.62 ± 96.54	457.94 ± 136.32	9.637	<.001
**β_2_MG** (mg/L)	2.48 ± 0.82	6.34 ± 8.03	3.899	<.001
**CYS-C** (mg/L)	1.66 ± 0.51	2.15 ± 0.53	2.638	<.001

BUN: urea nitrogen; CREA: creatinine; UA: uric acid; β2MG: β2-microglobulin; CYS-C: cystatin-C; Before-T: Before treatment; After-T: After treatment. Statistical methods: iPaired sample *t*-test.

### 3.5. Independence verification of different factors

**Figure 2.**
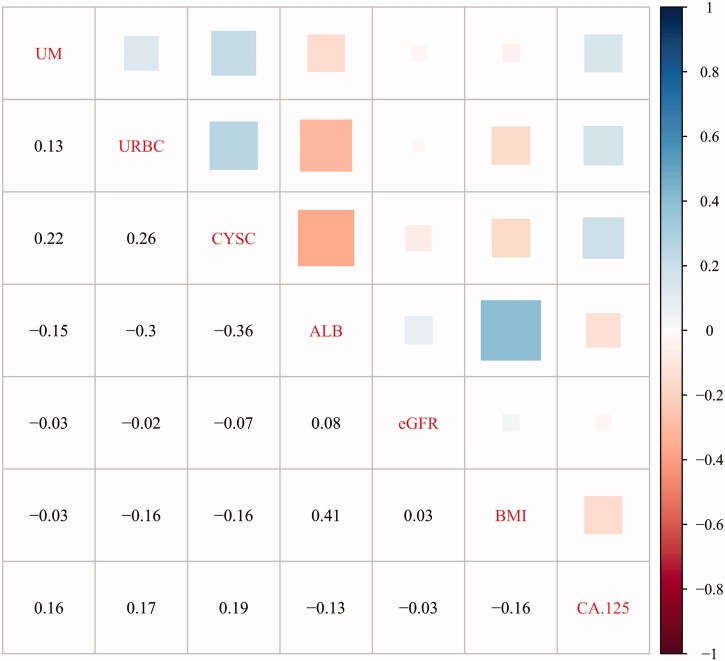
Independence verification of different factors (UM: microalbuminuria; URBC: Hematuria; CYS-C: cystatin C; BMI: body mass index).

Seven different factors were obtained through the above comparison. Spearman’s correlation analysis was used to verify the independence of the seven factors (Figure 2). In the positive correlations, the correlation between BMI and ALB was the highest (*r* = 0.41); In the negative correlations, the correlation between ALB and CYS-C was the highest (r= −0.36); The correlation between all variables was low, (r< ±0.5). Four continuous variables (BMI, CYS-C, ALB, and eGFR) were independent of each other (Table 5). There was no significant multicollinearity between the four continuous variables (VIF < 5, R^2^<1).

**Table 5.** The result of multicollinearity analysis.

	U Std. Coefficients		Std. Coefficients	Statistics		R^2^	Durbin- Watson
Factors	*Β*	*S.E*	*β*	Tol	VIF		
BMI	−0.024	0.006	−0.167	0.875	1.143	0.479	0.908
CYS-C	0.190	0.019	0.437	0.862	1.160		
ALB	−0.028	0.003	−0.351	0.910	1.099		
eGFR	0.000	0.000	−0.020	0.922	1.084		

U Std. Coefficients: Unstandardized Coefficients; Std. Coefficients: Standardized Coefficients; Tol: tolerance; VIF: variance inflation factor; eGFR: estimated glomerular filtration rate; CYS-C: cystatin-C. Statistical methods: Multicollinearity analysis.

### 3.6. Logistic regression analysis of the different factors between the two groups

To determine the predictors for AKI incidence in patients with PTB during anti-TB treatments, seven parameters (microalbuminuria, hematuria, CYS-C, ALB, eGFR, BMI, and CA-125) were included in the binary logistic regression.

According to the OR, four parameters were high-risk factors for AKI in patients with PTB during anti-TB treatments: UM-microalbuminuria (OR = 3.038, 95%CI 1.168–7.904), URBC-hematuria (OR = 3.656, 95%CI 1.325–10.083), CYS-C (OR = 4.416, 95%CI 2.296–8.491), and CA-125 (OR = 3.93, 95%CI 1.436–10.756). On the other hand, ALB (OR = 0.741, 95%CI 0.650–0.844) was a protective parameter against the development of AKI (Table 6).

**Table 6.** Logistic regression analysis of the difference factors.

*Factors*	*Β*	*S.E*	*Waldχ^2^*	*p-value*	*OR*	*95%CI*	
						low	up
UM	1.111	0.488	5.187	.023	3.038	1.168	7.904
URBC	1.296	0.518	6.271	.012	3.656	1.325	10.083
CYS-C	1.485	0.334	19.821	.000	4.416	2.296	8.491
ALB	−0.3	0.067	20.243	.000	0.741	0.650	0.844
eGFR	−0.012	0.007	2.791	.095	0.989	0.975	1.002
BMI	−0.135	0.102	1.74	.187	0.874	0.716	1.068
CA-125	1.369	0.514	7.097	.008	3.93	1.436	10.756

BMI: body mass index; UM: microalbuminuria; URBC: Hematuria; eGFR: estimated glomerular filtration rat; CA-125: cancer antigen 125; OR: odds ratio; CI: confidence interval. Statistical methods: Binary logistics regression analysis.

The risk of AKI in patients with PTB during anti-TB treatments was calculated by the following the binary logistic regression equation: *ln(p/1 − p)* = 8.244 + 1.111(with microalbuminuria) + 1.296*(with Hematuria) + 1.485*(CYS-C value) + 1.369*(with positive CA-125) − 0.3*(ALB value). In the equation, *p* represents the probability of AKI in patients with PTB during anti-TB treatments.

### 3.7. Nomogram construction and validation

**Figure 3.**
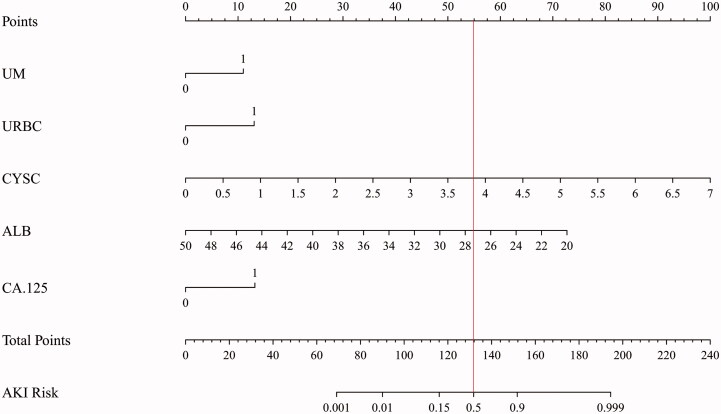
The Nomogram plot of logistic regression (UM: microalbuminuria; URBC: Hematuria; ALB: Albumin; 1: positive; 0: negative).

A prognostic nomogram for the early recognition of AKI in patients with PTB before anti-TB treatment was constructed using the binary logistic regression results, and points were assigned to the predictive factors according to their regression coefficients (Figure 3). As shown in the nomogram plot, patients with PTB who had microalbuminuria, hematuria, and higher values of CYS-C and CA-125 were more likely to develop AKI during anti-TB treatment. Nevertheless, patients with PTB who had higher ALB values were at a lower risk for the development of AKI. By summing the total score and locating the score on the whole point scale, the development of AKI can be predicted for patients with PTB before anti-TB treatment.

**Figure 4.**
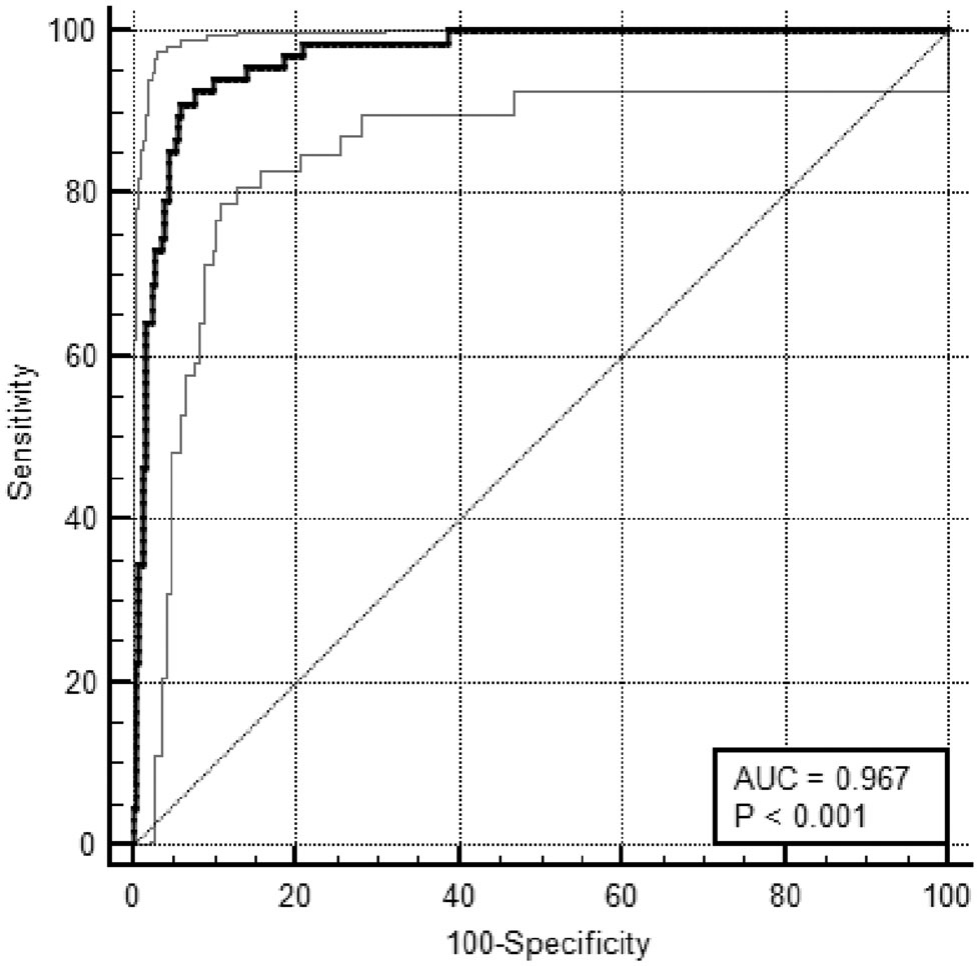
The ROC Curve of the discrimination (AUC: 0.967; 95%CI: (0.941–0.984); Youden index; *J*: 0.850; Sensitivity: 91.04%; Specificity: 93.95%).

To evaluate the discrimination of the prediction model, the C-index was calculated by the bootstrapping technique. The predicted values of the model plotted the ROC curve. The C-index was 0.967, and the AUC of the ROC curve was AUC = 0.967, (95%CI = 0.941–0.984) Youden index *J* = 0.850, sensitivity was 91.04%, specificity was 93.95% (Figure 4).

**Figure 5.**
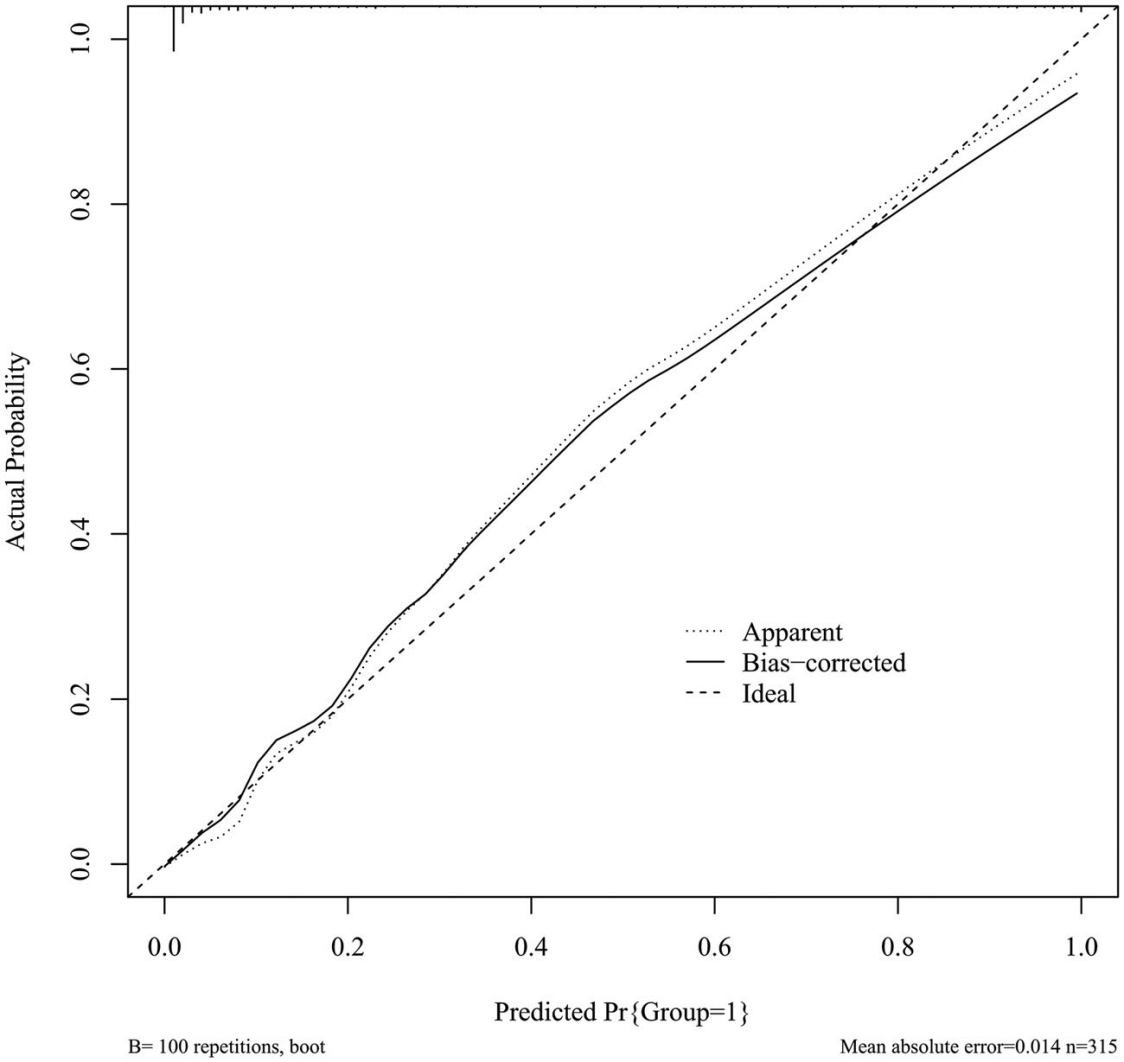
The Calibration curve of the prediction model (Mean absolute error = 0.014; Mean squared error = 0.00054; Quantile of absolute error = 0.049).

To evaluate the calibration of the prediction model, the calibration curve was plotted by Hosmer-Lemeshow analysis. The prediction model had good calibration (Figure 5).

### 3.8. Characteristics of immunological data of 63 patients with PTB

The immunological data of 19 patients with PTB who had AKI and 49 patients with PTB who did not had AKI were measured in this study. The numbers of CD3^+^ T cells in the AKI group were significantly higher than those in the control group; CD4 + T cell/CD8+ T cell in the AKI group were significantly higher than those in the control group. The values of IgG and IgA in the AKI group was also significantly higher than those in the control group (*p* < .05) (Table 7).

**Table 7.** The immunological data of 63 PTB patients.

Variable	Control Group	AKI Group	Total	Statistics(t)	*p-value*
**n**	49	19	68		
**CD3^+^**	66.07 ± 14.06	73.15 ± 10.01	68.05 ± 13.37	2.004	.049
**CD3^+^/CD8^+^**	24.22 ± 9.31	25.15 ± 12.04	24.48 ± 10.06	0.339	.735
**CD3^+^/CD4^+^**	39.26 ± 13.49	45.58 ± 14.48	41.03 ± 13.96	1.697	.094
**CD16^+^/CD56^+^**	14.22 ± 9.64	13.60 ± 9.22	14.05 ± 9.46	0.242	.809
**CD19^+^**	11.81 ± 7.13	8.49 ± 5.80	10.88 ± 6.90	1.807	.075
**CD4^+^/CD8^+^**	2.00 ± 1.14	3.16 ± 1.73	2.32 ± 1.42	3.230	.002
**IgG**	11.49 ± 2.76	19.15 ± 11.91	13.63 ± 7.46	4.262	<.001
**IgA**	2.36 ± 0.91	3.02 ± 1.4	2.54 ± 1.10	2.295	.025
**IgM**	1.01 ± 0.43	1.08 ± 0.73	1.03 ± 0.52	0.460	.647
**C3**	1.03 ± 0.23	0.95 ± 0.24	1.00 ± 0.23	1.295	.200
**C4**	0.26 ± 0.09	0.23 ± 0.07	0.25 ± 0.09	1.113	.270

CD3^+^: CD3 T cells; CD4^+^: CD4 T cells; CD8^+^: CD8 T cells; CD16^+^: CD16 natural killer cells; CD56^+^: CD56 natural killer cells; CD19^+^: CD19 B cells; IgG: immunoglobulin G; IgA: immunoglobulin A; IgM: immunoglobulin M; C3: complement 3; C4: complement 4. Statistical methods: independent sample *t*-test.

## 4. Discussion

Acute kidney injury (AKI) is not a rare complication during anti-TB treatment in some special populations [7]. Renal function impairment that fails to recover within 7 days will progress to acute kidney disease (AKD) [14]. Therefore, the prediction model to identify the risk for AKI is significant.

Chronic diseases such as diabetes, hypertension, and viral hepatitis are common causes of damage to kidney function [15]. More importantly, diabetes mellitus is a significant risk factor for latent TB [16]. A total of 184 patients with PTB who had an underlying disease were not included in our study to avoid mixed effects. After comparing the clinical data of 371 patients with PTB, seven factors (microalbuminuria, hematuria, CYS-C, albumin, eGFR, BMI, and CA-125) were acquired to develop the predictive model. The independence of factors is verified before the logistic analysis. According to the binary logistic regression analysis, four parameters (microalbuminuria, hematuria, CYS-C, and CA-125) were high-risk factors for AKI in patients with PTB during anti-TB treatments, while ALB was a protective parameter against the development of AKI.

As an endogenous inhibitor of cysteine proteinases, CYS-C plays an essential role in controlling protease activity in immune system regulation, and antiviral, and antimicrobial activities. Eventually, CYS-C is partially reabsorbed and decomposed in proximal tubular cells [17, 18]. In this study, there was a relatively large dissociation between creatinine and CYS-C. These results indicated that, in the early stages of kidney injury, compared with serum creatinine or eGFR, CYS-C has a higher sensitivity to predict early renal damage.

Although studies have confirmed that the value of CA-125 is related to the severity of PTB [19], serum CA-125 is beneficial in the differentiation between active and inactive PTB [20]. However, the mechanism of CA-125 elevation after TB infection is still unclear. Previous studies have confirmed that CA-125 is associated with most mesothelial membranes. CA-125 can also bind to the mesothelial cell surface glycoprotein mesothelin, indicating a potential role in cell adhesion in health and disease [21]. We believe that the level of CA-125 can reflect the degree of inflammation caused by TB.

TB can lead to malnutrition, and poor nutritional status may predispose individuals to TB. ALB and BMI are direct indicators that reflect the nutritional status of patients. Cell-mediated immunity is the critical host defense against *M. tuberculosis.* Malnutrition is a significant risk factor for immune system abnormalities in patients with TB [22]. Both innate resistant and adaptive immune cells play specific roles in the development of AKI [23]. Consequently, severely malnourished patients with PTB have a higher risk for developing AKI.

Urine microalbumin is considered an important marker of preclinical nephropathy [24]. In this study, approximately 50% of patients with PTB who had AKI had hematuria before anti-TB treatment [25,26]. After comparing the immunological indices, we confirmed that patients with PTB who had AKI had high numbers of CD3^+^ T cells. The values of CD4^+^T cell/CD8^+^ T cell, IgG, and IgA in the AKI group were significantly higher than those in the control group. The natural infection process of *M. tuberculosis* is complex. First, *M. tuberculosis* invades alveolar macrophages, replicates in macrophages, spreads to macrophages, myeloid dendritic cells, and neutrophils recruited from the surrounding areas, and finally triggers adaptive immunity [27]. The tubercle bacillus produces excessive tumor necrosis factor after infection and has extreme mitochondrial reactive oxygen species (ROS) through the mitochondrial-endoplasmic reticulum circuit, triggering the programmed necrosis of macrophages. AKI is associated with increased oxidative damage [28]. Superoxide radical anions, peroxides, and hydroxyl radicals can oxidize biomolecules and membranes, affect organelle function, and eventually induce renal tubular cell injury [29]. Active TB is frequently associated with a substantial increase in serum IgA levels. In addition, the ratio of T cells is positively correlated with serum IgA levels. The deposition of immune complexes in the kidney leads to renal dysfunction [30].

Confusingly, we do not yet know the exact cause of AKI in patients with PTB, whether the deposition of immune complexes produced by TB infection damages the glomerulus, whether anti-TB drugs (such as rifampicin) can cause tubular or interstitial damage, or whether both play an essential role is unknown. At present, we can make it clear that some patients with PTB have a higher risk of AKI during anti-TB treatment, and nephrotoxic drugs and special tests (such as coronary angiography) need to be avoided [31].

Nomograms were introduced to the medical field by J.L. Henderson in 1928. Nomograms can be used to predict the probability of the outcome of the regression model [32]. As shown in the nomogram plot (Figure 3), patients with PTB who had microalbuminuria, hematuria, and a higher value of CYS-C, and CA-125 were more likely to develop AKI during anti-TB treatment. Nevertheless, patients with PTB who had higher ALB levels were at a lower risk of developing AKI. By summing the total score and locating the score on the full point scale, the development of AKI can be predicted for patients with PTB before anti-TB treatment. Patients with PTB who had a total score of 132 have a 50% risk of developing AKI after anti-TB treatment. ROC curve and Hosmer-Lemeshow analyses were used to evaluate the prediction model discrimination and calibration of the prediction model. According to the analysis mentioned above, we believe that the initial treatment of patients with PTB who had malnutrition, microalbuminuria, hematuria, CA-125 positivity, and a high CYS-C value should not receive anti-TB immediately, supposing patients with PTB fail to improve their nutritional status and fail take measures to protect their kidney function in advance. In that case, they have a greater risk of AKI after the start of anti-TB treatment.

## 5. Limitations

In this study, the proportion of AKI in patients with PTB was higher than that reported in relevant clinical studies. The individuals included in our study were hospitalized patients with more severe pulmonary lesions, leading to statistical bias. This study failed to collect the long-term outcomes of patients with AKI, and the relevant factors affecting the long-term effects of patients will be statistically verified in the future. The mechanism of AKI in patients with PTB needs to be further studied to provide a theoretical basis for clinical prevention and treatment.

## 6. Conclusions

Microalbuminuria, hematuria, ALB, elevated CYS-C, and CA-125 positivity are predictive factors for AKI in patients with PTB during anti-TB treatments. Through the predictive nomogram based on five predictive factors, the risk of AKI in patients with PTB during anti-TB treatments can be determined earlier. Such an application is helpful for timely intervention to improve the patient’s prognosis.

## Data Availability

The datasets used and analyzed during the current study are available from the corresponding author on reasonable request.

## Abbreviations

CEA: carcinoembryonic antigen
CA-125: cancer antigen 125
CA19-9: cancer antigen19-9
BMI: body mass index
RBC: red blood cell
HB: hemoglobin
NE: neutrophile granulocyte
LYM: lymphocyte
MON: monocyte
PLT: blood platelet
USG: urine specific gravity
UCR: urine creatinine
Hematuria: URBC
TBIL: total bilirubin
DBIL: direct bilirubin
TP: total protein
ALB: albumin
ALT: alanine aminotransferase
AST: aspartate aminotransferase
AL P: serum alkaline phosphatase
GGT: gamma-glutamyl transpeptidase
LDH: lactate dehydrogenase
CK: creatine kinase
ACE: angiotensin converting enzyme
HCY: homocysteine
BUN: urea nitrogen
CREA: creatinine
UA: uric acid
β2MG: β2-microglobulin
CYS-C: cystatin-C
eGFR: estimated glomerular filtration rate
CRP: C-reactive protein
ESR: erythrocyte sedimentation rate
T-SPOT: T-SPOT.TB
CD3^+^: CD3 T cells
CD4^+^: CD4 T cells
CD8^+^: CD8 T cells
CD16^+^: CD16 natural killer cells
CD56^+^: CD56 natural killer cells
CD19^+^: CD19 B cells
IgG: immunoglobulin G
IgA: immunoglobulin A
IgM: immunoglobulin M
C3: complement 3
C4: complement 4
Statistical methods: independent sample *t*-test
